# A real‐world survey of the diagnostic journey, current treatment, and burden of mild cognitive impairment or dementia due to Alzheimer's disease in China

**DOI:** 10.1002/alz70860_096170

**Published:** 2025-12-23

**Authors:** Jiawei Xin, Hanxi Zhang, Shuang Cai, Jinnan Li, Guanshen Dou, Brenda Botello Estrada, Chloe Walker, Xiaochun Chen

**Affiliations:** ^1^ Fujian Medical University, Fuzhou, Fujian, China; ^2^ Eli Lilly And Company, Shanghai, Shanghai, China; ^3^ Eli Lilly and Company, Bracknell, Berkshire, United Kingdom; ^4^ Adelphi Real World, Bollington, Cheshire, United Kingdom

## Abstract

**Background:**

We aimed to describe the diagnostic journey, current treatment, caregiving needs, and burden of patients with mild cognitive impairment (MCI) and Alzheimer's disease (AD) dementia in China.

**Method:**

Data were drawn from the Adelphi Real World AD Disease Specific Programme™, a cross‐sectional survey of neurologists and their patients with MCI/AD across China, between 2024/02–2024/05. Neurologists completed patient record forms for their next (up to) nine consecutively consulting MCI/AD patients, capturing the diagnostic journey, current treatment, and caregiver support. Patients were invited to provide a self‐completion questionnaire, reporting on delays to first seeing a doctor and quality of life via the EQ‐5D visual analogue scale. Disease severity was defined using Mini Mental State Examination (MMSE) score: MCI/mild AD (20‐28) or moderate/severe AD (0‐19). Analyses were descriptive.

**Result:**

Ninety‐three neurologists reported data on 774 patients (mean±standard deviation age 69.2±8.3 years, body weight 64.2±11.4 kg, 49.1% female), of whom 192 self‐completed a patient survey. Of 699 patients with an available MMSE, 52.5% were currently MCI/mild AD and 47.5% moderate/severe AD. Median (interquartile range [IQR]) time between symptom onset and first consultation was 18.1 (8.4, 40.3) weeks, with 34.7% of patients experiencing moderate/severe AD by their initial consultation. Most patients (80.0%) were not diagnosed at initial consultation, with median (IQR) time from first consultation to diagnosis taking 4.4 (1.9, 12.9) weeks. Few patients (18.8%) had a biomarker confirmed diagnosis (mainly cerebral spinal fluid [11.2%] and amyloid positron emission tomography [7.7%]). Acetylcholinesterase Inhibitors (66.0%), *N*‐methyl‐D‐aspartate antagonist (33.5%), and nootropics (26.4%) were the most common current treatments, and long‐lasting effect was the top (37.1%) identified area for improvement. Among those with MCI/mild AD, 79.4% received non‐professional care (NPC) and 2.7% required professional care (95.2% and 15.7% for moderate/severe AD, respectively). On average, NPC partners spent 36.7±39.5 hours/week for MCI/mild AD and 47.3±39.6 hours/week for moderate/severe AD, and spouse/partner was the most frequent NPC partner. Mean EQ‐5D‐VAS score was 71.9±11.1 for those with current MCI/mild AD and 63.7±16.6 for moderate/severe AD.

**Conclusion:**

Further improvement is needed in early detection and accurate diagnosis of MCI/AD to lessen the burden to patients, care partners, and society.

